# *In situ* growth of well-dispersed CdS nanocrystals in semiconducting polymers

**DOI:** 10.1186/1556-276X-8-382

**Published:** 2013-09-09

**Authors:** Anna Maria Laera, Vincenzo Resta, Emanuela Piscopiello, Valerio Miceli, Monica Schioppa, Anna Grazia Scalone, Francesca Di Benedetto, Leander Tapfer

**Affiliations:** 1 ENEA, Technical Unit for Material Technologies, Brindisi Research Centre, Strada Statale 7, Appia km 706, Brindisi, 72100, Italy

**Keywords:** Chemical synthesis, Nanocrystals, Hybrid nanocomposites, 78.67.Bf, 79.60.Fr, 81.16.Be, 81.07.Pr, 83.80.Uv

## Abstract

A straight synthetic route to fabricate hybrid nanocomposite films of well-dispersed CdS nanocrystals (NCs) in poly[2-methoxy-5-(2'-ethyl-hexyloxy)-1,4-phenylene vinylene] (MEH-PPV) is reported. A soluble cadmium complex [Cd(SBz)_2_]_2_·MI, obtained by incorporating a Lewis base (1-methylimidazole, MI) on the cadmium bis(benzyl)thiol, is used as starting reagent in an *in situ* thermolytic process. CdS NCs with spherical shape nucleate and grow well below 200°C in a relatively short time (30 min). Photoluminescence spectroscopy measurements performed on CdS/MEH-PPV nanocomposites show that CdS photoluminescence peaks are totally quenched inside MEH-PPV, if compared to CdS/PMMA nanocomposites, as expected due to overlapping of the polymer absorption and CdS emission spectra. The CdS NCs are well-dispersed in size and homogeneously distributed within MEH-PPV matrix as proved by transmission electron microscopy. Nanocomposites with different precursor/polymer weight ratios were prepared in the range from 1:4 to 4:1. Highly dense materials, without NCs clustering, were obtained for a weight/weight ratio of 2:3 between precursor and polymer, making these nanocomposites particularly suitable for optoelectronic and solar energy conversion applications.

## Background

**Figure 1:**
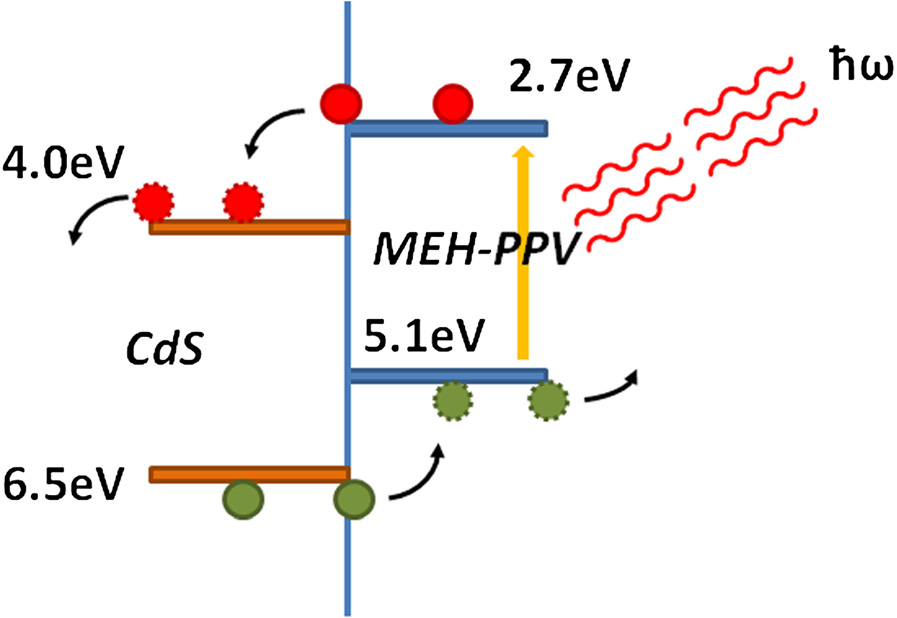
**Schematic energy level diagram for the CdS/MEH-PPV hybrid nanocomposite.** With energy levels in eV relative to vacuum.

Organic–inorganic hybrid nanocomposites have attracted great interest over recent years for their extraordinary performances due to the combination of the advantageous properties of organic polymers and the unique size-dependent properties of nanocrystals (NCs) [1]–[3]. Furthermore, the interaction between the matrix and the nanocrystalline fillers provides new peculiar features including mechanical [4], optical [5] and electrical properties [6] to the nanocomposite. In particular, the use of conjugated polymers has been intensively investigated in view of the efficient photo-induced charge transfer between conjugated polymers and semiconductor NCs [7]. In solar cells with nanocomposite materials as active layer, ‘bulk heterojunction’, the NCs act as electron acceptors (n-type material) and the polymer acts as electron donor (p-type material). Among the inorganic semiconductors, CdS is considered a promising material for its strong visible light absorption and, recently, a power conversion efficiency of 1.17% has been reached using the poly[2-methoxy-5-(2'-ethyl-hexyloxy)-1,4-phenylene vinylene] (MEH-PPV) as matrix [8]. Figure 1 shows the band structure of the CdS/MEH-PPV inorganic–organic hybrid system.

Efficient photoconductivity requires not only efficient charge separation but also efficient transport of the carriers to the electrodes without recombination, in that sense, the morphology of nanocomposite being crucial in providing suitable paths for both electron and hole towards the appropriate electrode [7]. The NC network must be homogeneous so that each negative charge can efficiently hop to another NC in the direction of the internal field, this requirement being a complex issue when NCs are dispersed in polymeric matrices. The main difficulty is due to the high surface-to-volume ratio of NCs that tend to form agglomerate to lower their surface energy. Furthermore, the addition of a dense network of NCs to polymers can significantly alter the mechanical properties of the resulting nanocomposite material compromising the advantageous properties of organic semiconductor such as the easy processability [9].

The nanocomposite is frequently gained by solution blending, i.e. dispersion of NCs in polymer solutions that can be dried under vacuum or can be used to obtain thin films by spin-casting (solvent evaporation) [10]. During these procedures, the NCs form microsized aggregates and cannot be separated from each other. As a consequence, nanocomposites have been commonly prepared by synthesis of the inorganic NCs *in situ*, for instance in solution, where the solvent is a monomer and the nanocomposite is then prepared through *in situ* polymerization [11,12]. Alternatively, the inorganic NCs can be synthesized inside polymer matrices through the thermolysis of suitable precursors. Recent works of our research group have demonstrated that cadmium thiolates are promising materials for the *in situ* synthesis of nanocrystalline CdS [13]–[18]. Using unimolecular precursors, as cadmium thiolates, it is possible to overcome any problem, occurring in the other chemical methods, such as the low temporal stability of reagents, the inhomogeneity of multicomponent mixing and the intrinsic high reactivity and toxicity of the precursor used. Furthermore, unimolecular precursors guarantee the stoichiometry control of thermolytic process. Unfortunately, cadmium thiolates, having a polymeric structure, are insoluble in typical organic solvents; so, it is not possible to homogeneously disperse them in polymeric matrices, and the thermolysis process induces the growth of CdS NCs with a disordered distribution.

In this work, we present a straight convenient approach to synthesize well-dispersed CdS NCs in pristine MEH-PPV using a cadmium complex obtained by incorporating a Lewis base (1-methylimidazole, MI) on the cadmium bis(benzyl)thiol [19], the [Cd(SBz)_2_]_2_·MI adduct (for the sake of brevity CBzMI). The latter proves to be highly soluble in the most common organic solvents. Solutions of the polymer MEH-PPV and the cadmium complex allow to obtain large area composite films by spin coating, making the proposed technique not expensive and ideal to fabricate optoelectronic devices.

## Methods

All the reagents used to synthesize the precursor and the polymer were purchased from Sigma-Aldrich S.r.l., Milan, Italy, and used without further purification. All the nanocomposites were prepared using the pristine polymer MEH-PPV with a number of average molecular weight (Mn) of 70,000 to 100,000.

The synthesis of Cd(SBz)_2_ was conducted using the commercial salt cadmium nitrate hexahydrate (9 mmol) as starting reagent. After the dissolution of cadmium salt in ethanol, an aqueous solution of ammonium hydroxide (25%) was added and, as a consequence, the starting opaque solution became clear. When the benzyl mercaptan (18 mmol) was added in the reaction vessel, the desired product precipitated in quantitative yield and it was isolated from the solution by filtration.

The soluble complex [Cd(SBz)_2_]_2_·MI was performed suspending the thiolate Cd(SBz)_2_ and adding dropwise 1-methyl imidazole (MI) until a clear solution was obtained. The product was purified by crystallization from toluene, cooling the solution to −18°C. Thermogravimetric analysis (Netzsch-Gerätebau GmbH STA429 simultaneous thermal analyzer, Selb, Germany) allowed to confirm the general formula of the obtained Lewis base-derived complex [Cd(SBz)_2_]_2_·MI in which the stoichiometric ratio between thiolate and MI is 2:1 [13].

The precursor/polymer composite films were produced by spin coating on glass slides, silicon wafers and copper grids from the solutions of [Cd(SBz)_2_]_2_^.^MI and MEH-PPV in chloroform with a respective weight/weight ratio of 1:4, 2:3 and 4:1, respectively. The same procedure was realized using an inert polymer as polymethyl methacrylate (PMMA) for comparative aims. The spin speed and time were set at 1,500 rpm and 10 s, respectively, in order to obtain uniform and smooth polymer films. For all samples, the thermolysis process was performed at temperatures of 175°C, 185°C and 200°C for 30 min with a reproducible controlled ramp and in nitrogen atmosphere to avoid possible oxidation of NCs surface.

Optical properties of the annealed samples, by means of a Xe lamp (LC8 Hamamatsu, Hamamatsu City, Shizuoka, Japan) and a HR460 monochromator (Jobin Yvon, Kyoto, Japan), were investigated on chloroform solutions obtained by the samples deposited on glass. UV-visible transmission were performed in order to evaluate the absorbance of the specimens as ln(1/T). Photoluminescence (PL) spectra were acquired on the same chloroform solutions with a Varian Cary Eclipse Fluorometer, Palo Alto, CA, USA, (excitation wavelength, 330 nm).

In order to investigate the formation of CdS NCs, their structural properties, crystallographic structure and size, X-ray diffraction measurements were performed on the annealed samples on glass substrates using a Philips PW1880 X-ray diffractometer, Almelo, The Netherlands, having a CuKα radiation source (3 kW).

Using a TECNAI F30 transmission electron microscope (TEM), FEI, Hillsboro, OR, USA, operating at 300 kV and point-to-point resolution of 0.205 nm, the structural characterization of the samples deposited on carbon-coated copper grids was also executed. Finally, rheological measurements were carried out by a parallel plate rheometer stress tech HR at 200°C. Samples of MEH-PPV and CdS/MEH-PPV nanocomposites, with a relative weight ratio of 1:4, were prepared by casting of solution in chloroform to obtain 1-mm thick films in order to evaluate the influence of CdS NCs inclusion on MEH-PPV film mechanical properties.

## Results and discussion

### Thermolytic process and thermogravimetric analysis

The thermolytic process to obtain CdS NCs is described by the following scheme:

(1) $${\left[\mathrm{Cd}{\left({\mathrm{SCH}}_{2}C_{6}H_{5}\right)}_{2}\right]}_{2}\mathrm{MI}\to 2\;\mathrm{Cd}{\left({\mathrm{SCH}}_{2}C_{6}H_{5}\right)}_{2}+\mathrm{MI}\to 2\;\mathrm{CdS}+{2\;C}_{6}H_{5}{\mathrm{CH}}_{2}{\mathrm{SCH}}_{2}C_{6}H_{5}$$

**Figure 2:**
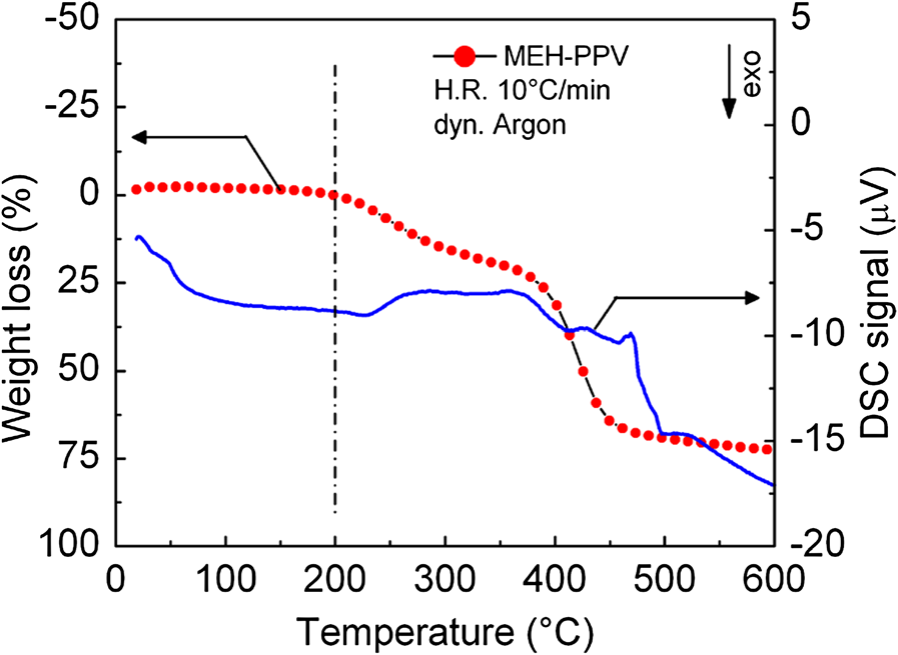
**TG and DSC signals of MEH-PPV film.** In argon atmosphere and recorded in the temperature range 25°C to 600°C (heating rate, 10°C/min).

Thermogravimetric analysis, reported elsewhere [13], shows that the imidazole ligand is broken when the temperature reaches about 100°C, while the remaining metal bis(thiolates) decompose in a second step forming cadmium sulphide when temperature reaches 180°C. Our studies demonstrated that annealing temperatures of about 180°C to 200°C are required for the formation of CdS NCs. However, this finding implies that the thermal stability of the polymer at these annealing temperatures must be also assured. In fact, the thermal stability of polymers is one of the most important properties for both processing and application [20]. Thermogravimetric (TG) and differential scanning calorimerty (DSC) signals of MEH-PPV film show the polymer degradation in the temperature range 25°C to 600°C, in inert atmosphere (Figure 2). The first weight loss on TG curve in the temperature range 200°C to 300°C is associated to the decomposition of MEH group (first broad exothermic peak on DSC curve). The weight loss occurred at higher temperature is associated to a double exothermic peak and corresponds to the decomposition of PPV structure. Consequently, our results show that MEH-PPV films are still stable at the used annealing temperatures and the polymer decomposition becomes critical at temperatures >200°C consistent with the decomposition of MEH side groups and PPV backbone at low and high temperatures, as reported in the literature [21].

### Optical spectroscopy analysis

**Figure 3:**
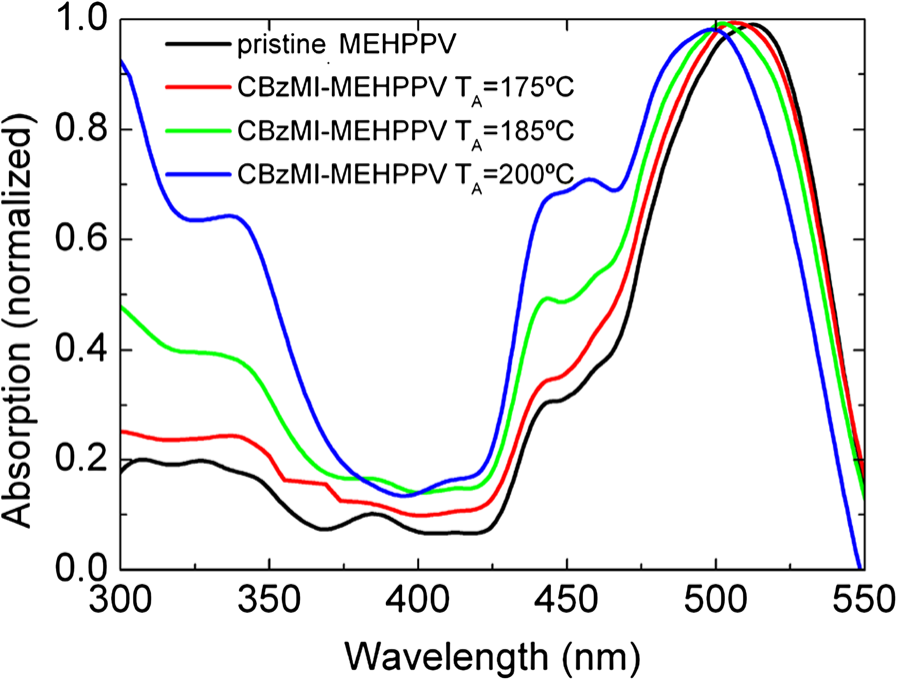
**Absorption spectra of non-annealed and annealed samples.** At 175°C, 185°C and 200°C with a precursor/polymer weight/weight ratio of 1:4.

The absorption spectra of the [Cd(SBz)_2_]_2_·MI/MEH-PPV samples with a weight/weight ratio of 1:4 recorded before and after the annealing process are shown in Figure 3. The optical absorption spectrum of [Cd(SBz)_2_]_2_·MI-doped MEH-PPV prior to annealing is characterized by an absorption band at approximately 500 nm due to π-π* transition of conjugated chain in MEH-PPV and by an absorption edge at approximately 370 nm related to the precursor contribution [13,18]. Whereas, for the samples annealed at 175°C, 185°C and 200°C, an absorption band at 350 nm gradually grows in intensity as the temperature increases. This band can be attributed to the first optically allowed transition between the electron state in conduction band and the hole state in the valence band, its increase in intensity indicating an increase of the NCs concentration. The MEH-PPV absorption band remains peaked at 500 nm, indicating the absence of ground state charge transfer. Values of NCs size estimated using the general theoretical model of the Brus equation are reported in Table 1 and state that NC size gradually increases in the considered range of temperature below the threshold of the Bohr exciton radius.

**Table 1:** CdS NC size calculated from absorption data

**Annealing temperature (°C)**	**Absorption edge (nm)**	**Band gap absorption (eV)**	**CdS NC size (from Brus equation [**[22]**]) (nm)**
175	359	3.50	2.8
185	368	3.36	3.1
200	384	3.22	3.5

**Figure 4:**
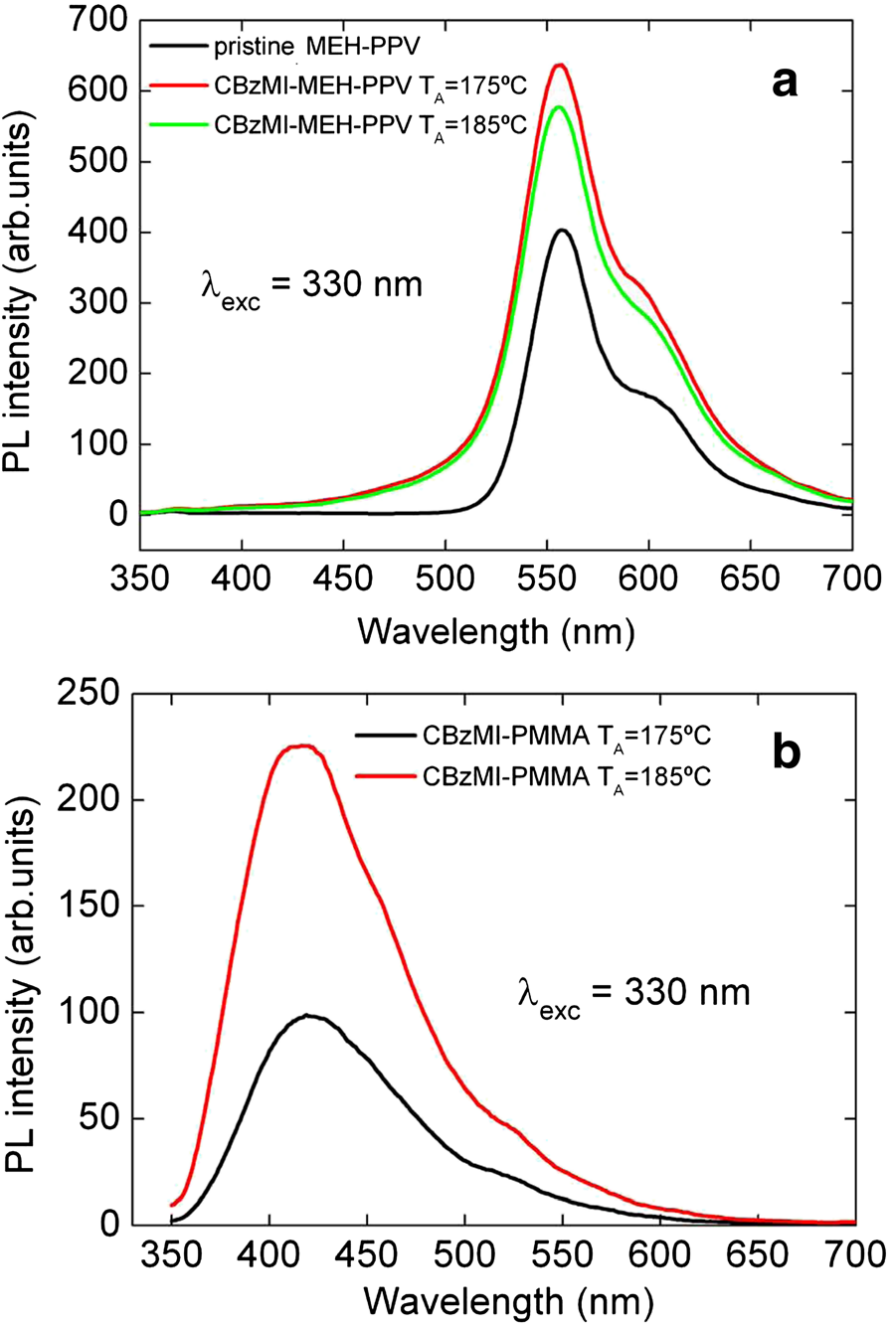
**PL spectra of CdS NCs.** In MEH-PPV **(a)** and in PMMA **(b)** grown at 175°C and 185°C (excitation wavelength 330 nm), respectively.

In Figure 4a, the PL spectra of CdS/MEH-PPV nanocomposites, obtained at 175°C and 185°C, for the samples with a weight/weight ratio of 1:4 exclusively show the emission band of conjugated polymer around 550 nm. As expected, the PL peaks of CdS NCs appear totally quenched inside MEH-PPV because of the overlapping between polymer absorption and CdS emission. Furthermore, the polymer fluorescence appears highly quenched and broader, when annealing temperature increases, in consequence of NCs concentration growth [10]. The well-known emission peaks of pure MEH-PPV are located at approximately 580 and 625 nm (both noticeable in Figure 4a) and are ascribed to a single-chain (or intrachain) exciton emission and interchain (or aggregation or excimer) emission. The MEH-PPV luminescence quenching indicates that the annealing treatment promoted the aggregation of polymer chains, and the degree of aggregation increases as the annealing temperature increases [23]. The fact that no red shift of the emission spectra for the CdS/MEH-PPV occurs indicates that no aggregation of polymer chains is induced by incorporation of the NCs into the polymer matrix [24]. To complete the spectroscopic characterizations of CdS NCs, exactly alike thermolysis experiments were performed for comparison in PMMA that is optically transparent in the visible region, thus allowing a complete characterization of the NCs fillers. In PMMA, the PL emission shows a maximum at 420 nm for both CdS/PMMA nanocomposites obtained at 175°C and 185°C (Figure 4b) with a weight/weight ratio of 1:4. As derived by comparing the position of emission peak with literature data, CdS NCs average size in PMMA is 3 nm [25]. Although the nucleation and growth speed can be influenced in different ways by MEH-PPV and PMMA, it is possible to obtain interesting general information about the optical quality of CdS NCs by observing PL emission spectra in PMMA. The deconvolution of emission band allows to put in evidence two different signals: the first one, with a maximum at 420 nm, due to the emission from band edge, and the second one, in the range 520 to 560 nm, due to ‘shallow defect’. These reticular defects, mainly localized on the NCs surface, can be attributed to anionic insaturation [26,27]. In the literature, many examples of CdS NCs in which shallow defects play an important role are reported [28,29]. In our case, the intensity of emission from shallow defects is very low with respect to the emission band edge, indicating a good optical quality of synthesized CdS NCs.

### Microstructural analysis: X-ray scattering and transmission electron microscopy

**Figure 5:**
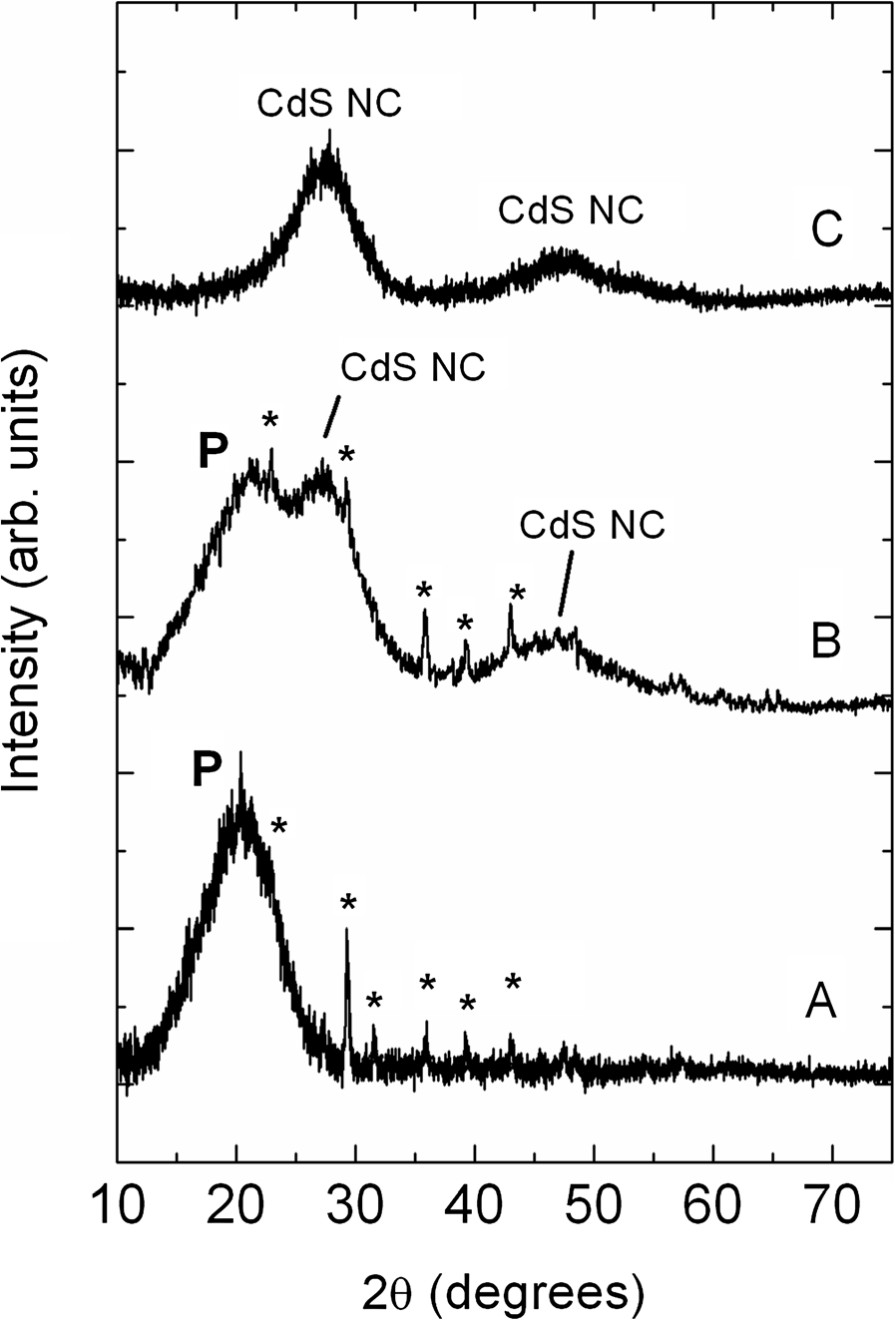
**X-ray scattering measurements (WAXS) of CdS/MEH-PPV nanocomposites.** Obtained at 185°C for samples with precursor/polymer weight/weight ratio of 1:4 (curve **B**) and 4:1 (curve **C**). For reference and comparison, the WAXS pattern of pristine MEH-PPV is also shown (curve **A**). The diffraction peaks labelled as ‘P’ and asterisk ‘*’are due to the crystalline nanodomains of the conjugated polymer.

The X-ray diffraction (wide angle X-ray scattering (WAXS)) measurements of CdS/MEH-PPV nanocomposites obtained at 185°C for the samples with a weight/weight ratio of 1:4 and 4:1 are shown in Figure 5. Curve A shows the WAXS pattern of the pristine MEH-PPV polymer (without of [Cd(SBz)_2_]_2_·MI precursors) exhibiting the broad polymer peak (labelled as P) and the characteristic weak Bragg peaks (denoted by asterisk ‘*’) that are related to the presence of nanodomains of mesomorphic order, i.e. crystallites of orthorhombic structure (local packing chains of MEH-PPV chains), as observed and reported in the literature [30,31]. In particular, the broad peak P corresponds to the interbackbone spacing (0.43 nm) in the direction normal to the coplanar phenylene rings, while the periodic angular peak distribution yields a lattice spacing of about 2.5 nm, and is in very good agreement with the bilayer spacing of the two neighbouring MEH-PPV chains (2.47 nm), i.e. MEH-PPV ethylhexyloxy side groups are interdigitated [32].

Curve B in Figure 5 shows the WAXS pattern of the CdS/MEH-PPV nanocomposites obtained after annealing at 185°C for the samples with a weight/weight ratio of 1:4. Here, besides the MEH-PPV diffraction peaks, broad X-ray peaks attributed to the formation of CdS nanocrystals are also observed. Also, curve C obtained for the samples with a weight/weight ratio of 4:1 shows the CdS nanocrystal peaks. However, in this case, the polymer peaks (P and the weak peaks of the polymer superstructure) are not observed or are too low to be experimentally observed due to the low polymer content.

In order to analyse the structure and particle size of the CdS nanocrystals, the experimental WAXS pattern of the sample with a weight/weight ratio of 1:4 (curve C) is compared to the simulated X-ray diffraction patterns for cubic (zinc blende) and hexagonal (wurtzite) CdS nanocrystals. For the sake of simplicity, here, we focus our comparison to curve C because in curve B, the polymer peak P is overlapped to the main CdS diffraction peak, but as can be easily seen, the conclusion and findings will be identical for curve B.

**Figure 6:**
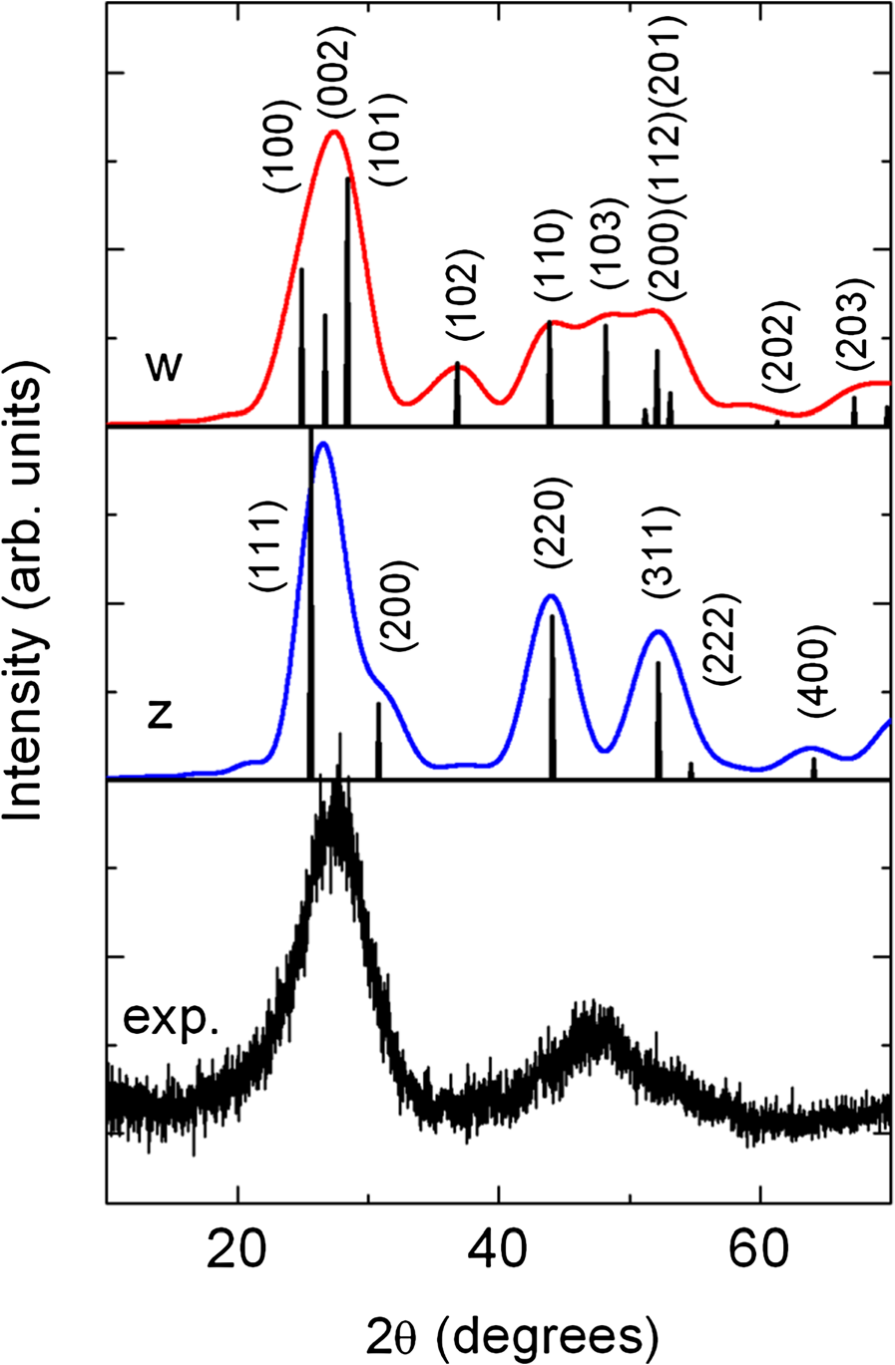
**Experimental WAXS pattern (curve C in Figure**5**) and calculated X-ray diffraction patterns.** For CdS nanocrystals of cubic (zinc blende, labelled as ‘z’) and hexagonal (wurtzite, ‘w’) crystallographic phase. The nanocrystals are assumed to be of spherical shape and having particle size (diameter) of 3 nm.

Figure 6 shows the experimental WAXS pattern that corresponds to curve C in Figure 5, and the calculated WAXS pattern of CdS nanocrystals of particle diameter of 3 nm of zinc blende (curve z) and wurtzite (curve w) crystallographic structure, respectively. The X-ray diffraction patterns are calculated using the model of Langford [33] and assuming particles of spherical shape and, for simplicity, without size dispersion. For comparison, together with the calculated patterns, the Bragg peaks are also shown (their angular position and relative intensities) in accordance with the ICDD cards for the cubic (PDF nr. 80–0019) and hexagonal (PDF nr. 80–0006) CdS phase [JCPDS-ICDD ©2000].

For this kind of polymer nanocomposite samples, it is not very easy to perform quantitative X-ray analyses; nevertheless, by comparing the calculated patterns with experimentally measured patterns, we find a much better agreement for the wurtzite phase of the CdS nanocrystals. This is particularly evident for the shape of the main diffraction peak (convolution of more Bragg peaks) at about 2*θ* = 27.6° and for the broad peak at about 2*θ* = 47°. Nevertheless, we cannot exclude the presence and coexistence of CdS nanocrystals of zinc blende phase within the hybrid nanocomposite.

**Figure 7:**
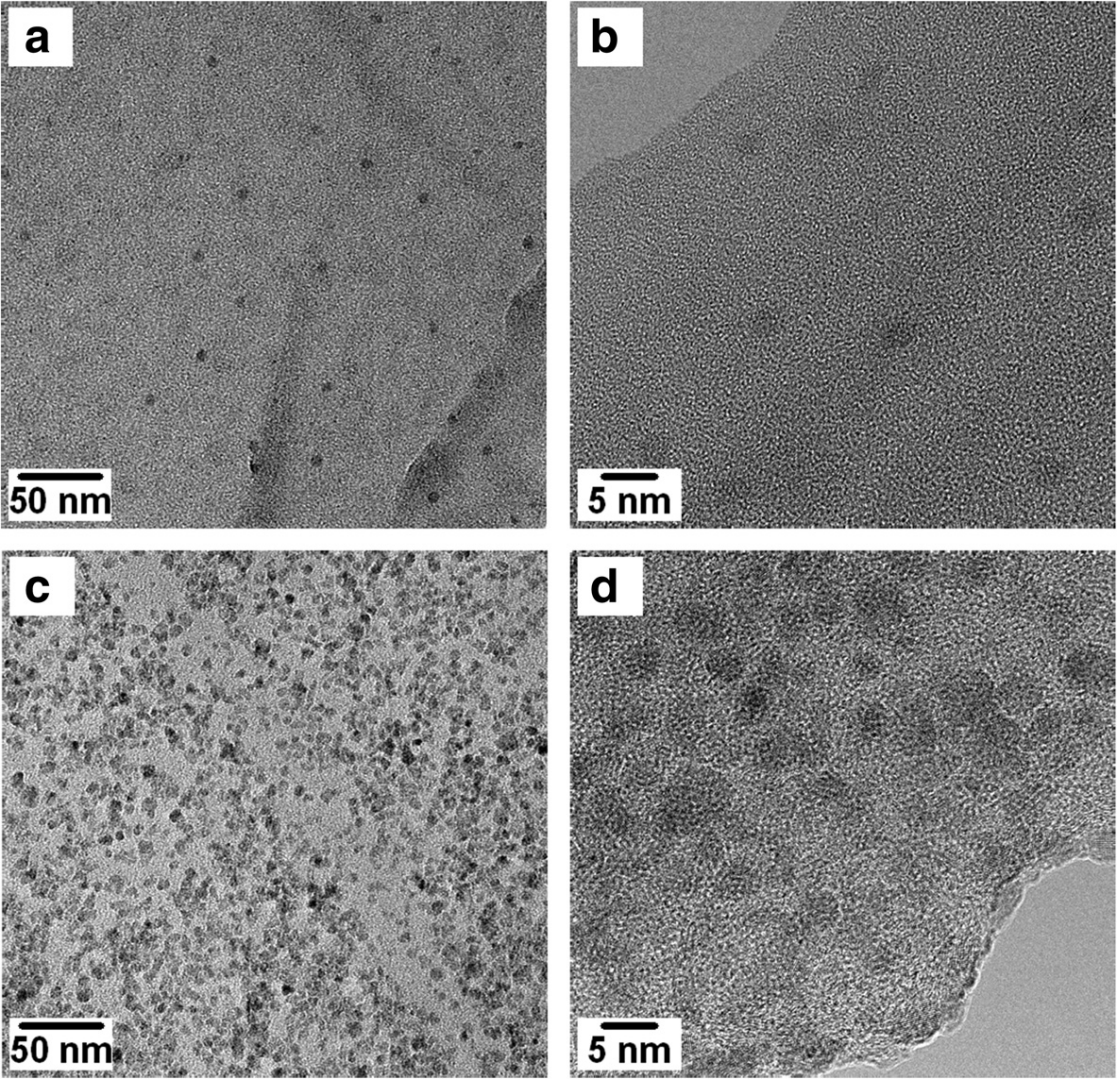
**TEM images of CdS/MEH-PPV nanocomposites.** Obtained at 185°C starting from a solution with a weight/weight ratio precursor/polymer of 1:4 **(a**, **b)** and from a solution with a weight/weight ratio precursor/polymer of 2:3 **(c**, **d)**.

In order to further investigate the structure of CdS/MEH-PPV nanocomposites, the thermolysis process was performed directly on thin composite films deposited on carbon-coated copper grids for TEM observations. In Figure 7a,b, TEM images of CdS/MEH-PPV nanocomposites obtained at 185°C, for the sample with a weight/weight ratio of 1:4, show the formation of CdS NCs with a regular spherical shape and a very homogeneous distribution in MEH-PPV matrix. Nevertheless, the density of nanocomposite is very low for application in photovoltaic and light detection devices; in fact, the average distance among the CdS NCs is above 50 nm. Further experiments were performed using a respective weight/weight ratio between precursor and polymer of 2:3. This ratio percentage allows to obtain a dense regular network of CdS NCs inside MEH-PPV without evident agglomerates, as shown in Figure 7c,d. Here, it should be noted that experiments performed using a weight/weight ratio precursor/polymer of 4:1 allowed to obtain only microsized crystals of CdS.

**Figure 8:**
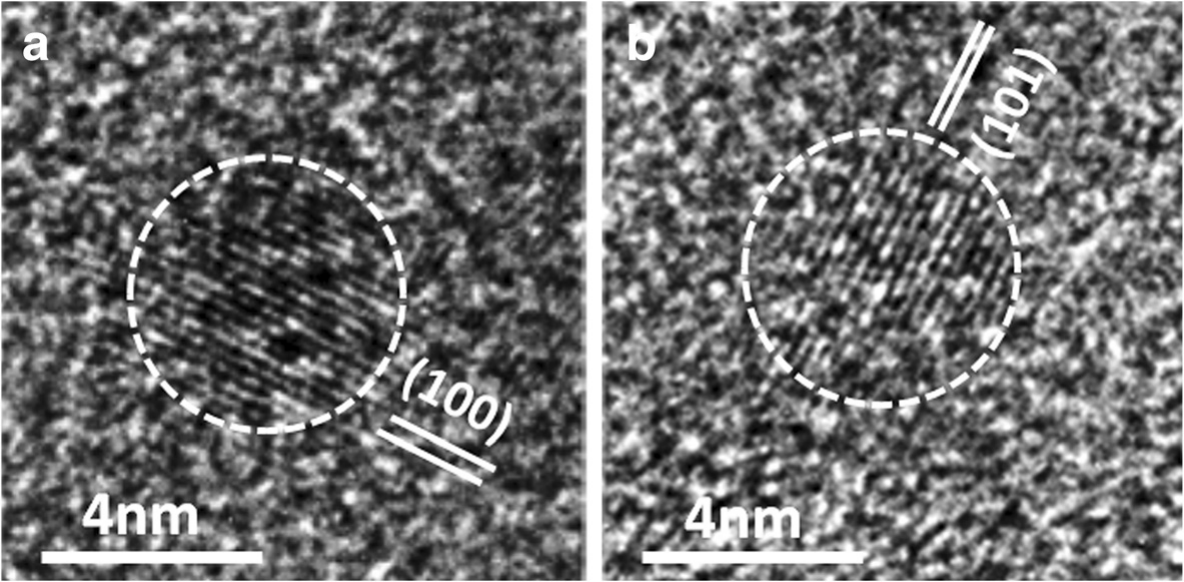
**High-resolution TEM images of CdS NCs (marked by dashed lines).** Exhibiting (100) and (101) lattice fringes in **(a)** and **(b)**, respectively. The NCs are of almost spherical in shape and diameter between 3 and 4 nm.

Figure 8a,b shows the high-resolution images of single CdS NCs. The nanoparticles are highlighted (marked by dashed line) in order to better visualize the CdS NCs within the polymer matrix. The (100) and (101) lattice fringes of the wurtzite phase of CdS are well observed and are distinct from the amorphous matrix. The particles are of spherical shape, and the average diameter is about 3 to 4 nm.

### Rheological properties of the nanocomposite films

**Figure 9:**
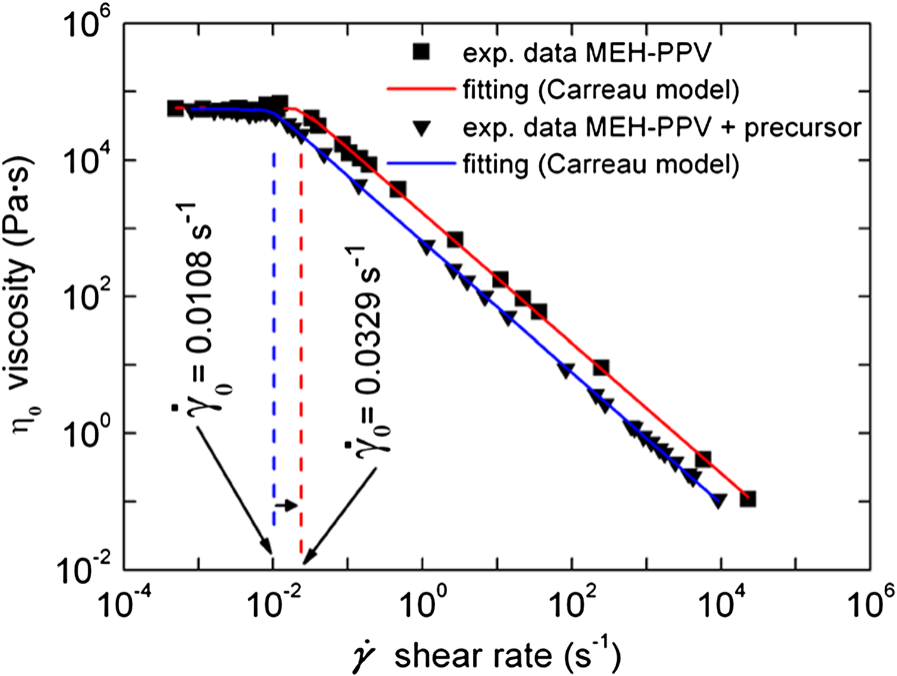
**Rheological measurements of pristine MEH-PPV and CdS/MEH-PPV nanocomposites.** With a weight/weight ratio between precursor and polymer of 1:4 carried out at 200°C.

Figure 9 shows the viscosity of MEH-PPV and nanocomposite CdS/MEH-PPV with a relative weight ratio of 1:4 depending upon the values of shear rate. At low shear rate, both materials show a Newtonian behaviour, while at high shear rate, the viscosity linearly decreases as a non-Newtonian fluid described in Carreau model [34]. The fitting of the experimental data using the Carraeu model yields a viscosity of *η*_0_ = 5.745 × 10^4^ Pa·s and *η*_0_ = 5.498 × 10^4^ Pa·s, for the pristine polymer and CdS/MEH-PPV nanocomposite, respectively. In addition, the transition from a Newtonian to a non-Newtonian behaviour occurs at a viscosity of (a) *η*_0_ = 4.09 × 10^4^ Pa·s and shear rate ${\dot{\gamma }}_{0}=0.0329\;s^{-1}$, and (b) *η*_0_ = 5.213 × 10^4^ Pa·s and shear rate ${\dot{\gamma }}_{0}=0.0108$, respectively.

These results indicate that the inclusion of NCs into the polymer matrix does not significantly alter the polymer resistance to deformation. Applications in the field of large-area, flexible, low-cost solar cells require to preserve the nanoflow material rheology to allow the developing of fabrication process based on spinning or soft moulding lithography.

The rheological measurements complete the characterization of prepared CdS/MEH-PPV hybrid nanocomposites. All data acquired by absorption spectroscopy, X-ray diffraction and TEM show the growth of CdS NCs with a regular spherical shape, a narrow size distribution and a homogenous dispersion inside the polymer. The use of 1-methylimidazole ligand to improve the solubility of Cd(SBz)_2_ has allowed to obtain clear solutions of the complex [Cd(SBz)_2_]_2_·MI and MEH-PPV in chloroform, suitable to prepare thin solid film using the cheap and easy technique of spin coating. The CdS NCs size grows from 2.8 to 3.5 nm in the temperature range 175°C to 200°C, demonstrating a slow and controlled diffusion of Cd(SBz)_2_ molecules inside the matrix. Nevertheless, the polymer prevents the formation of microsize aggregate by limiting the diffusion capability of CdS NCs.

The presented synthetic strategy allows a good control of NC size and distribution within the polymer matrix as required for the application in photovoltaic cells.

## Conclusions

An *in situ* synthetic route for the realization of hybrid polymer/nanocomposite materials was presented. We demonstrated that the soluble metal thiolate derivative [Cd(SBz)_2_]_2_·MI, obtained using 1-methylimidazole as cadmium ligand, is a suitable starting material to grow CdS NCs in semiconducting polymeric matrices. We found that the precursor decomposition and the subsequent NCs nucleation and growth start at temperatures below 200°C, namely already at 175°C and in relatively short time (30min), the temperature lowering being crucial for avoiding possible damage or deterioration of the matrix. Such a result allows extending the range of suitable matrices to thermally soft polymers such as MEH-PPV towards the fabrication of organic–inorganic nanocomposite materials for optoelectronics and light harvesting.

The structure of [Cd(SBz)_2_]_2_·MI also helps in obtaining a homogeneous spatial dispersion of the molecule itself inside the polymer promoting the formation of a highly uniform network and well-dispersed NCs. The weight ratio of the precursor to the polymer directly determines the number density of the NCs as well as the coverage uniformity, the optimal value being 2:3.

The synthetic route did not significantly alter the polymer resistance to deformation, further demonstrating the applicability in the field of large-area, flexible, low-cost solar cells production via spinning or soft moulding lithography.

## Competing interests

The authors declare that they have no competing interests.

## Authors' contributions

AML carried out the precursor synthesis and the polymer nanocomposite synthesis by thermolysis and drafted the manuscript. VR carried out the optical spectroscopy experiments and participated in the thermolysis processes. EP carried out the TEM experiments and image analysis. VM carried out the rheological experiments. MS carried out the TGA and DSC measurements. AGS participated in the polymer nanocomposite synthesis by thermolysis. FDB carried out the X-ray measurements and participated in the nanocomposite preparation. LT conceived of the study, participated in its design and coordination and participated in drafting the manuscript. All authors read and approved the final manuscript.
